# Guidelines for repeated measures statistical analysis approaches with basic science research considerations

**DOI:** 10.1172/JCI171058

**Published:** 2023-06-01

**Authors:** Lutfiyya N. Muhammad

**Affiliations:** Department of Preventive Medicine, Division of Biostatistics, Feinberg School of Medicine, Northwestern University, Chicago, Illinois, USA.

## Introduction

A study design with repeated measures data occurs when each experimental unit has multiple dependent variable observations collected at several time points. Often longitudinal data (1, 2) are used interchangeably to describe repeated measures data. This study design also includes two or more experimental groups, and each experimental group receives varying levels of experimental conditions. The experimental group variable is treated as the primary independent variable in the statistical analysis. Study designs with repeated measurements are common in basic science research. A systematic review of preclinical animal research studies found that approximately 50% of studies within biomedical research domains of toxicology and brain trauma reported a study design with repeated measurements (3). Planned statistical analyses for this type of research design should incorporate all observed repeated measurements to evaluate the research hypotheses. However, often statistical analyses aggregate repeated measures data by computing an average for each experimental unit.

When the repeated measurements are aggregated, ANOVA is generally applied. Using an ANOVA with aggregated repeated measurements violates the key ANOVA assumption of independence. Repeated measurements are correlated observations, given that they are observed from the same experimental unit. Repeated measurements collected closer in time are more correlated than measurements collected further in time (4). Correlation within an experimental unit is ignored when repeated measurements are aggregated. This violation of independence leads to biased analysis results and incorrect interpretations (1).

An extension of an ANOVA is a repeated measures ANOVA. A repeated measures ANOVA accounts for the correlation within and between experimental groups (5) along with the time of the measurements (time point 1, time point 2, etc.). Similar to an ANOVA, time is treated as a categorical variable (6) rather than a continuous variable in a repeated measures ANOVA. Assumptions of the repeated measures ANOVA should be carefully considered when determining if it is an appropriate statistical approach. Statistical method alternatives to a repeated measures ANOVA are applicable when repeated measures assumptions are not met. The purpose of this Viewpoint is to provide guidance on statistical analysis options for repeated measurements within the context of basic science. Analysis guidance is given considering various assumptions and repeated measures data structures.

## Repeated measures ANOVA requirements and considerations

The assumptions of a repeated measures ANOVA are that the continuous dependent variable is approximately normally distributed, the categorical independent variable (e.g., experimental group) has three or more levels, no outliers in any of the repeated measurements, and sphericity (constant variance across time points). All assumptions are required to be met for a repeated measures ANOVA to be an appropriate statistical analysis approach. In a review of 58 preclinical animal studies (7), it was found that the checking of assumptions of repeated measures analyses were not accurately reported. Specifically, assumptions related to variance were not described. The sphericity assumption is a strong assumption that may not be accurate when there are repeated measurements. Mauchly’s test can determine if the sphericity assumption is reasonable (8), and there are adjustments such as the Huynh-Feldt and Greenhouse-Geisser corrections that can account for the violations of the sphericity assumption (9–11). These adjustments are easy to apply with statistical software. Results from analyses that utilize these approaches should be stated in descriptions of statistical methods. Transforming the continuous dependent data so that they are approximately normally distributed or using Friedman’s test (nonparametric version of the repeated measures ANOVA) can be done when the normality assumption is invalid.

A repeated measures ANOVA requires a balanced number of repeated measurements for each experimental unit. Due to this requirement, experimental units with missing measurements are completely excluded from the analysis (i.e., complete case analysis), which results in the sample size decreasing and the type II error increasing (12, 13). By excluding experimental units with missing data, the statistical power also decreases. Sample sizes are typically smaller in basic science research, and any reduction in the sample size due to missing data can greatly effect the results (6).

Missing data can occur in basic science for various reasons. The reporting of missing data in basic science research is not standard practice as it is in clinical trials (14). Therefore, the description of statistical analysis methods in peer-reviewed publications could include the sample size of the complete cases only (7) or may not describe if an imputation technique was used for the missingness. Simple imputation approaches such as mean imputation and last observation carried forward are considered as remedies for missing data and alternatives to the complete case approach (2). Another missing data technique is multiple imputation. Instead of replacing the missing observation with a single value, like in simple imputation, multiple imputation replaces the missing observation many times, utilizing distributional properties and information from the observed data. However, these imputation approaches have their limitations, given small sample sizes that are commonly observed in basic science research (12, 15). The type of missingness should be determined when selecting an imputation technique. The three types of missingness are missing at random, missing completely at random, and missing not at random. Missing at random describes the scenario when the missing data are independent of the unobserved measurement. Missing completely at random is missing data that are independent of the observed and unobserved measurements. Missing not at random is data that are missing due to the unobserved measurement. Multiple imputation is applicable when missing at random occurs.

## Alternative statistical approach: mixed-effects models

A mixed-effects model is a statistical model with fixed and random effects. Estimates from mixed-effects models provide interpretations that are experimental unit specific. This means that the interpretations focus on the expected change of the dependent variable for an experimental unit or cluster of experimental units (1, 12). Fixed effects are parameters that do not vary, such as experimental group and sex of animal. Random effects in a model can be random slopes, random intercepts, or both. Random effects allow for multiple sources of variability within the data to be captured in the mixed-effects model. Sources of variability can be the result of complex study designs with clustering and hierarchical structures. Litter size (16) and clusters of gene profiles (17) are examples within basic science that are often treated as random effects. There is not a strict sphericity assumption for mixed-effects models. Instead, mixed-effects models can account for different covariance structures so that sources of variability are appropriately included (4, 13, 18).

Within the mixed-effects model framework there are linear mixed-effects models, generalized linear mixed models, and nonlinear mixed-effects models (19). Like an ANOVA and repeated measures ANOVA, the dependent variable must be continuous and approximately normally distributed for a linear mixed-effects model. Generalized linear mixed models and nonlinear mixed-effects models are extensions of linear mixed-effects models. Generalized linear mixed models are used for discrete dependent variables such as cell counts that are measured repeatedly. Nonlinear mixed-effects models can handle complex, nonlinear dependent variables. Tumor growth within mice (20) and pharmacokinetics of animals (21) have been modeled using nonlinear mixed-effects models.

Mixed-effects models incorporate time and the imbalance of repeated measurements across experimental units differently than ANOVA and repeated measures ANOVA. Mixed-effects models have the flexibility to treat the time of repeated measurements as a continuous variable or as a categorical variable. Various covariance structures can be used to construct mixed models with unequal timing between repeated measurements across experimental units (4). Mixed-effects models can include experimental units in the analysis, even with missing measurements or with a different number of repeated measurements in comparison to the remaining experimental units. The ability to handle an imbalance of measurements across experimental units is an advantage in comparison to repeated measures ANOVA.

Performance of mixed-effects models have been assessed under various scenarios of small sample sizes (12) and clustering sizes (22). Results from simulation studies of the mixed-effects models have showed that estimates were least biased when model assumptions were valid and denominator degrees of freedom adjustments were applied. Examples of denominator degrees of freedom adjustments are Kenward-Roger and between-within (22). Determining the validity of model assumptions can be difficult with small sample sizes. Distribution assumptions for the models are commonly assessed using histogram and Q-Q plots.

## Comparison of statistical approaches using simulated data

A simulated data example comparing results from ANOVA, repeated measures ANOVA, and a linear mixed-effects model is in Table 1. Published average body weights in grams and standard deviations of female C57Bl/6J mice (23) were used to generate normally distributed data for 3 groups. Body weights were monitored using dual energy x-ray absorptiometry. Body weight averages and standard deviations from three time points (week 5, week 9, and week 13) were used to produce data for 10 mice per group. Larger body weights in group 3 were generated to ensure that there were body weight differences across the groups. Several body weight measurements in all groups were removed from weeks 9 and 13 to simulate missing data. A total of 10 measurements were removed among the three groups. The removal of 10 body weight measurements caused there to be 80 body weight measurements that were not missing.

The sample sizes and number of measurements used for each statistical approach are included in Table 1. In order to utilize ANOVA, body weights had to be aggregated by computing an average for each mouse. The body weight average per mice allowed for there to be only one measurement per mouse. For the repeated measures ANOVA, only 21 mice with all measurements at week 5, week 9, and week 13 were included in the analysis. All 80 nonmissing body weight measurements from the 30 mice (10 mice per group) were used in the linear mixed-effects model. Each mouse was treated as a random effect in the linear mixed-effects model, so that the variability within and across groups could be captured in the model. The covariance structure in the linear mixed-effects model was a first-order autoregressive structure, such that measurements at adjacent time points had higher correlation than measurements at nonadjacent time points.

The *F* value and corresponding *P* value from each statistical approach are reported in Table 1. The *F* value from an *F* test was used to determine if there were statistically significant body weights differences across the 3 groups. The ANOVA results were not able to detect statistically significant differences across the groups. In addition, an ANOVA cannot determine whether there are group differences at each time point because an ANOVA is not able to account for time, like a repeated measures ANOVA and mixed effect model. The repeated measures ANOVA and linear mixed-effects model were able to detect statistically significant differences across the 3 groups. Despite the *P* values of both approaches being less than an α level of 0.05, the *P* value from the linear mixed-effects model was smaller than the repeated measures ANOVA. The repeated measures ANOVA and linear mixed-effects model were able to determine which groups differ. Due to having multiple statistical comparisons among the groups, the Tukey-Kramer *P* value adjustment was used to determine statistical significance. The linear mixed-effects model was the only approach that was able to detect a statistically significant difference between groups 2 and 3 at week 5.

## Conclusions

The appropriate statistical methods for repeated measurements must be applied to make accurate scientific interpretations from basic science research studies. Aggregating repeated measurements and applying an ANOVA is not suitable given the violation of the independence assumption. The assumptions of a repeated measures ANOVA should be assessed thoroughly, and decisions need to be made regarding how to handle an imbalance of repeated measurements if applicable. The mixed-effects model framework provides an alternate solution when there is an imbalance of repeated measurements, a complex study design, and/or a dependent variable that is not continuous. Software that carries out statistical analyses, including GraphPad Software, can be used to apply the repeated measures analysis approaches described in this Viewpoint. When there is uncertainty regarding which repeated measures analysis is the best fit, it is important to consult with a statistician. Statisticians can provide advice at any stage of the research study (24), but there is preference to consult with a statistician in the planning stage of a study.

## Figures and Tables

**Table 1:**
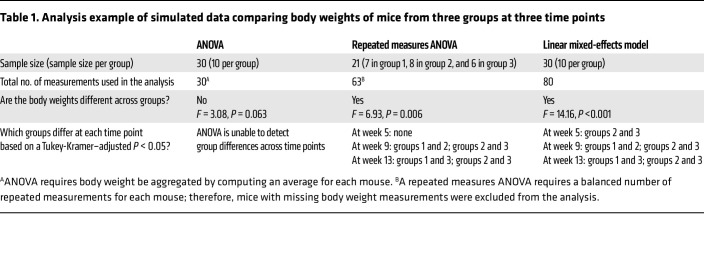
Analysis example of simulated data comparing body weights of mice from three groups at three time points

## Version 1 06/01/2023

Electronic publication
